# Low dose naltrexone for fibromyalgia: case series demonstrating pain relief and other health benefits

**DOI:** 10.3389/fpain.2026.1731455

**Published:** 2026-07-02

**Authors:** Adrienne Junek, Mya Anderson

**Affiliations:** 1 Department of Family Medicine, University of Ottawa, Ottawa, ON, Canada; 2 Bachelor of Science Honors, University of Ottawa, Ottawa, ON, Canada

**Keywords:** chronic pain, fibromyalgia, functional medicine, hormesis, integrative medicine, low dose naltrexone, naltrexone

## Abstract

Fibromyalgia is a chronic pain disorder with multiple proposed etiologies. Pharmacological treatments approved for fibromyalgia are few and not always effective; therefore, some practitioners use off-label medications such as low dose naltrexone (LDN). This case series presents a retrospective chart review of patients who were prescribed LDN for fibromyalgia at an integrative medicine clinic over a 2-year period. Outcomes assessed included pain relief, other health benefits, side effects and prescription habits. Of 10 patients identified, LDN provided substantial pain relief for 8 patients (−4.7/10 points, average −3.75/10 for all 10 patients, *p* < 0.001). These same patients also reported other important health benefits such better quality of life in 6 patients, reductions in pain flares in 5 patients, improved sleep in 4 patients, improved cognitive functioning in 2 patients, and cessation of other pain medications in 4 patients. Two patients discontinued LDN due to side effects, which in both cases was increased pain. LDN was well tolerated overall, although reported side effects included increased pain, vivid dreams, insomnia and irritability. LDN's proposed mechanism involving hormesis at opioid receptors and its implications for customized dosing in clinical care and research trials are discussed. While not all patients respond to LDN, this medication shows promise as an effective and safe intervention and deserves to be considered as a treatment recommendation for fibromyalgia.

## Introduction

Fibromyalgia is a chronic pain disorder involving central sensitization that can affect the whole body. Symptoms typically include chronic widespread pain with associated brain fog, fatigue, headaches and insomnia. Fibromyalgia disproportionately affects women and comorbidities commonly include headaches, irritable bowel syndrome, anxiety, depression and post-traumatic stress disorder (PTSD) including sexual trauma. Patients with fibromyalgia often need to reduce work schedules, physical activity and forego hobbies and social outings due to their symptoms.

Fibromyalgia is a clinical diagnosis and is principally cared for in a primary care setting (1). Recommended treatments include nonpharmacological management such as exercise, pacing and psychotherapy, as well as medications primarily used for neuropathic pain such as duloxetine, pregabalin, tramadol, medical cannabis and milnacipran (2, 3). Of these, only duloxetine and pregabalin are approved by Health Canada for fibromyalgia treatment.

Low dose naltrexone (LDN) has been used off-label to treat chronic pain disorders such as fibromyalgia (4). Naltrexone is typically dosed at 50 mg per day in the treatment of alcohol addiction (5) where it is understood to act by blocking opioid receptors. In contrast, low doses of naltrexone (range 0.1 mg to 6.0 mg) are postulated to involve hormesis (6, 7), whereby low doses of a compound can trigger an *adaptive* response while high doses of the same compound *no longer* trigger the same effect. This paradoxical response has been observed with opioid transmission in preclinical models (8) and is postulated to modulate endogenous opioid signalling (7). LDN has also been implicated in toll-like receptor signalling (9) as well as modulation of glial cell function and neuroinflammation (10); both of which have been proposed as some of the underlying mechanisms of fibromyalgia (11, 12). Naltrexone is generally considered safe at both high and low doses and has not been associated with hepatotoxicity or other serious side effects (13, 14). Common side effects include altered sleep and vivid dreaming; other occasionally reported side effects include headache, nausea, diarrhea, and dizziness (15, 16).

Initial small-scale studies have demonstrated potential efficacy of LDN (17, 18), including retrospective reviews of over 100 patients (19, 20). However, recent larger randomized control trials and observational trials have not always demonstrated significant pain relief (21–23). Recent meta-analyses interpreted benefits of LDN as effective (15, 16) or minimal (21) and high rates of discontinuation due to lack of therapeutic effect have been reported (22). Despite mixed findings in randomized control trials and meta-analyses, small case series continue to indicate the potential for LDN to offer substantial pain relief to some individuals (23), illustrating that ongoing study of this medication is warranted.

The current case series describes multiple beneficial health benefits including pain relief, tolerability and limited side effects with the use of LDN for fibromyalgia at an integrative medicine clinic. Furthermore, this study aims to share information on prescribing practices and their relevance to the hormetic mechanism of action of LDN to help future researchers understand the importance of slow dose titration and empower primary care providers to consider the use of LDN for fibromyalgia in their own practices.

## Methods

This study is a retrospective chart review of the clinical care and outcomes for patients who were prescribed LDN for fibromyalgia two integrative health clinics in Ottawa, Ontario, Canada. Patients were identified from a comprehensive review of all patients under the care of the attending physician between January 1, 2022 and April 1, 2024. Charts for all patients who were prescribed LDN for treatment of fibromyalgia were included for analysis; patients who were prescribed LDN for diagnoses other than fibromyalgia were excluded. Patients were diagnosed with fibromyalgia using the American College of Rheumatology criteria. LDN was often prescribed first-line due to patient preference or after discussion of the risks and benefits of both Health Canada-approved and off-label options.

Charts were reviewed for demographics, medical comorbidities, concurrent therapies and LDN prescription characteristics (starting dose, titration, final dose). The primary outcome was pain scores on a 0–10 numerical rating scale (NRS) from before and after treatment with LDN. Other patient-reported health benefits as well as side effects from LDN are reported as secondary outcomes. Follow up duration was defined as the interval from LDN initiation to last documented contact while on LDN, or up to discontinuation. For patients who discontinued immediately due to side effects, post-LDN pain scores were treated as unchanged from baseline. Pre & post treatment pain scores were compared using a paired 2-tailed t-test.

The study was conducted after several patients of the treating clinician received significant benefits from LDN and encouraged the author to review and publish data on the clinical effects of LDN. Potential reporting bias was thus mitigated by conducting a comprehensive search of all patient charts at the clinic over a 2.5 year period (estimated ∼200) to identify all patients who were prescribed naltrexone for fibromyalgia. Ethics approval for this case series was obtained via the University of Ottawa Office of Research Ethics and Integrity (H-09-23-9590).

## Results

### Patient demographics

A total of 10 charts were which included 9 females and 1 male, ages ranging from 21 to 65 years (Table 1). Pain scores using a numerical rating scale (NRS) from 0 to 10 were identified both before LDN initiation and at the last available follow-up. Patients were generally followed for long intervals, i.e., 1–2 years, except for 1 patient who was lost to follow up after 4 months and one patient who ceased use of LDN shortly after initiation due to side effects and stopped attending the clinic.

**Table 1:** Patient demographics & treatment summary.

Patient	Age sex	Comorbidities	Concurrent treatments	Initial dose	Final dose	Follow-up duration
A	45F	PTSD, myofascial back pain, opioid addiction history	cannabis, TPI, magnesium, GFD, relaxation exercises, somatic therapy	1.5 mg liquid	7 mg	10 months
B	34F	ADHD, migraines, GI issues	Cannabis, sumatriptan, acetaminophen, TPIs, probiotics, glutamine, B complex,	1.5 mg liquid	1.5 mg (stopped)	2 days
C	46F	IBS	cannabis, leflunomide, acupuncture, probiotics, glutamine, GFD/DFD, corn-free diet	1.5 mg capsule	6 mg BID	>2 years
D	62F	Headaches, tingling, stiffness	celecoxib	1.5 mg capsule	6 mg	4 months
E	52F	IBS	quercetin, probiotics, echinacea, GFD, DFD	0.5 mg capsule	2 mg (stopped)	<3 months
F	56F	Depression, pituitary tumor (hydrocortisone dependence)	Hydrocortisone, estrogen, progesterone, cabergoline, doxycycline, GFD	1.5 mg capsule	4.5–6 mg	>2 years
G	45F	IBS	Acupuncture, probiotics, glutamine, GFD, DFD, Egg free, Nut free	1.5 mg capsule	4.5 mg	>2 years
H	38M	PTSD/trauma, ADHD, depression, migraines, IBS	Amitriptyline, triptans, ketamine-assisted psychotherapy, methylphenidate, GFD, egg-free diet, probiotics	1.5 mg capsule	4.5 mg	1 year
J	21F	Bipolar disorder, anxiety	Amitriptyline, escitalopram, TPI, psychotherapy	1.5 mg capsule	3 mg	6 months
K	65F	IBS, asthma, allergies	Naproxen, GFD	0.5 mg liquid	4.5 mg	>1 year

PTSD, post-traumatic stress disorder, ADHD, attention deficit hyperactive disorder, TPI, trigger point injections, GFD, gluten free diet, DFD, dairy free diet.

### Prescription characteristics

LDN was prescribed as a first line therapy in 4 patients and as a second-line therapy in 4 patients who already took amitriptyline. LDN was initially prescribed as 1.5 mg nightly for 1 week; 3.0 mg for 1 week, then 4.5 mg thereafter. Dosing adjustments were made as needed based on clinical response: 2 patients opted to start LDN at 0.5 mg and increase by 0.5 mg increments and 2 patients increased the dose every few weeks instead of weekly to allow more time to adjust to side effects. The most common final dose of LDN was 4.5 mg, however 2 patients increased to higher doses for better pain relief and one patient returned to 3.0 mg as this produced better clinical outcomes than 4.5 mg. Two patients took LDN at 1.5 mg but discontinued due to side effects. Most patients purchased LDN from a compounding pharmacy, however, for financial reasons, 3 patients used cheaper 50 mg tabs which could be dissolved in 100 mL water (0.5 mg/mL) and dosed as liquid instead of capsules.

### Pain scores

**Figure 1:**
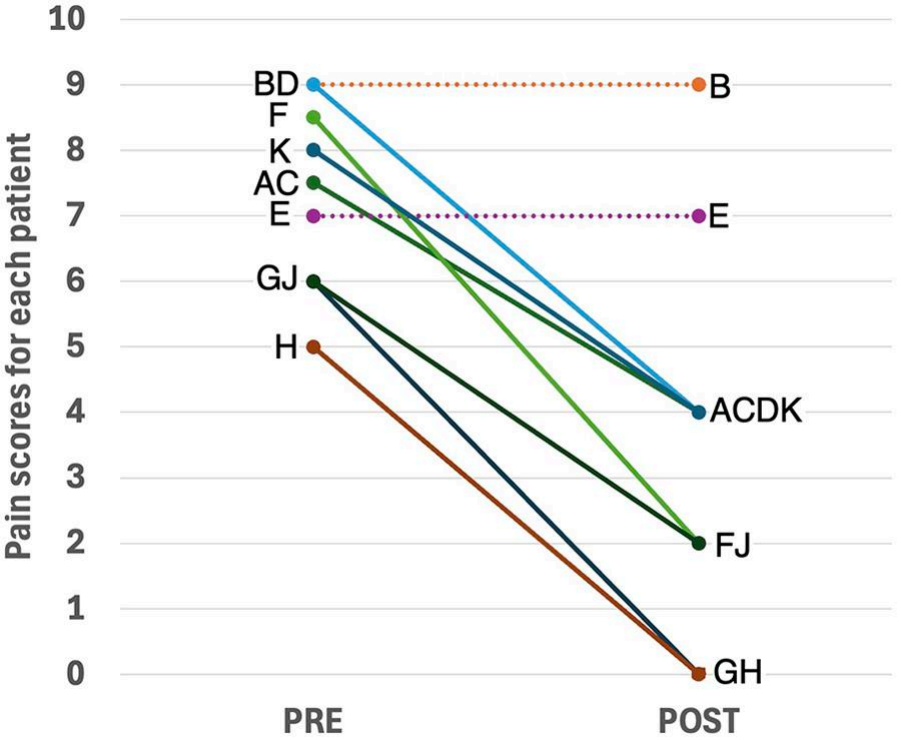
Pain scores before and after LDN. Two patients (A,C) had identical pre & post treatment pain scores and are graphed as a single line. Two patients (B,E) stopped LDN due to side effects; initial pain scores are extrapolated as unchanged from baseline (dotted lines).

Most (8/10) patients experienced pain relief from LDN, however 2 patients did not and ceased treatment with LDN (Table 2). Among all 10 charts reviewed, average daily pain scores decreased from 7.35/10 (range 5–9) before LDN to 3.6/10 (range 0–9) after LDN (Δ −3.75, range 0 to −6.5, *p* < 0.001). Among the 8 patient responders, average pain scores dropped from 7.2/10 (range 5–9) before LDN to 2.5/10 (range 0–4) after LDN (Δ −4.7/10, range −3.5 to −6.5; Figure 1). Onset of pain relief from LDN was variable from 1 to 2 days up to 1 month or longer with ongoing dose increases.

**Table 2:** Pain scores, treatment responses, health benefits and Side effects.

Patient	Pre-LDN pain score	Post-LDN pain score	Onset of relief	Other health benefits	Side effects
A	7–8/10	4/10	Weeks, improved over a few months	Better sleep, reduced brain fog, fewer spasms, reduced cravings, fewer flares	Irritability, insomnia
B	8–10/10	–	None (stopped after 2 days)	None	Migraines
C	7–8/10	3–5/10	1 month, more sustained benefit with BID dosing	Better sleep, return to work, able to exercise, avoided disability	None
D	9/10	4/10	4 months	Able to return to skiing, less exertional pain	None
E	7/10	–	None (stopped after a few weeks)	None	Increased pain
F	8–9/10	2–4/10	1 month (when reached 4.5 mg)	Tapered off amitriptyline and hydrocortisone, returned to cycling/workouts, improved mood	Vivid dreams
G	6/10	0/10	1–2 days	Returned to full-time work, resumed workouts, fewer flares	None
H	5/10	0/10	3 months	Brain fog gone, high energy, returned to full-time work, identified eggs as a trigger	None
J	6/10	2/10	1 week (when reached 3.0 mg)	Milder flares, tolerates injections better, tapered off amitriptyline	None
K	8/10	4/10	1–2 days	Better sleep, improved exercise tolerance, identified dairy as a trigger	Insomnia, vivid dreams, transient increased pain

### Secondary health outcomes

Among responders (*n* = 8), additional reported health benefits from LDN included the ability to return to work, leisure and social activities (6/8), less frequent/severe pain flares (5/8), improved sleep (4/8), and reductions in brain fog (2/8). Four patients tapered or stop other pain medications (4/8) which in all cases was amitriptyline; these were the only four taking other FBM medications at the time they started LDN (Table 2).

### Side effects

All charts documented whether side effects were present. Side effects were experienced by 5/10 patients and included increased pain (*n* = 3), vivid dreams (*n* = 2), insomnia (*n* = 2), and moodiness/irritability (*n* = 1) (Table 2). Two patients discontinued LDN due to side effects (increased pain) but one patient experienced transient increased pain with dose increases, followed by overall pain relief when she progressively increased the dose of LDN more slowly. Side effects were mild in all patients and tolerability was improved by increasing the dose in smaller increments (0.5 mg, K). Two patients experienced insomnia, which was circumvented by dosing LDN in the morning instead of the evening (A,K).

## Discussion

Most patients with fibromyalgia in this case series (8/10) responded positively to LDN with substantial overall pain reduction (−4.7/10 points). A direct causal effect of LDN on pain relief is further suggested as several patients experienced increased pain when they temporarily ceased LDN for various reasons. Many patients reported other health benefits such as return to work, exercise, hobbies, improved sleep, resolution of brain fog, and improved quality of life. LDN was successfully used both as a first-line therapy in 4 patients and as an add-on second line therapy for 4 patients who concurrently took amitriptyline: all 4 reduced or stopped amitriptyline entirely after LDN. Two patients ceased LDN due to side effects (increased pain); however, others continued to use LDN when the benefits outweighed side effects. Qualitative comments voluntarily offered and recorded in charts of LDN responders frequently reflected meaningful health benefits from LDN persisting for up to 2 years. Side effects overall were mild, transient, reversible upon cessation of LDN, and could be minimized with slower dose titration or smaller dose increments. We now highlight several factors which could contribute to the substantial observed treatment responses in this study which contrast with those of large-scale randomized control trials.

### Hormesis and custom dose titration

Hormesis refers to the existence of a “window of efficacy” of medication dosing for which there is an optimal dose or dose range for a medication, yet higher (or lower) doses may not produce the same therapeutic effect. The importance of customized dosing increments to maximize treatment responses to LDN has been reported previously (6) and was observed in several patients in this study. While 4.5 mg is the most common optimal dose, the range may be as wide as 0.1 mg to 6.0 mg, or beyond. The implementation of patient-specific dosing may have enabled better identification the dose of LDN that led to optimal pain relief and contributed to higher observed treatment response rates.

A hormetic mechanism of action could contribute to why some patients do not obtain therapeutic effects with LDN. For example, pain relief may not occur at all if the initiating dose is already beyond the window of efficacy; or could be lost if the initial dose is effective but is then increased beyond the window of efficacy. A prior study noted that some patients discontinued LDN as they progressively increased the dose due to “*loss of pain benefit*” (23, p16), an observation which was replicated in one of the patients in our chart review, consistent with this proposed mechanism.

Recognizing hormesis as a mechanism of LDN challenges the results of some prior studies which did not observe pain relief with LDN. Studies which incorporated dose titration or flexible patient specific dosing tended to report overall pain relief (6, 19, 20, 22), however two recent randomized control trials which initiated LDN outright at doses of 4.5 mg or 6.0 mg without dose titration or patient-specific dosing reported no overall pain relief (24, 25). This nuanced observation suggests that patient-specific dosing and/or dose titration may enhance the ability to obtain pain relief with LDN. A starting dose of LDN that is too high could contribute to lack of pain relief, increased pain as a side effect and high rates of dropout due to lack of therapeutic effect.

### Comprehensive care

The Canadian fibromyalgia guidelines (1) emphasize the importance of multi-modal care, incorporating both pharmacological and non-pharmacological treatments and promoting patient self-management. Patients in this case series generally self-referred voluntarily to the integrative medicine clinic, reflecting a group of motivated patients with self-management capacity.

The efficacy of LDN may also be improved in patients who concurrently address other individualized factors contributing to their pain. Multiple patients in this study followed a gluten-free diet or had other food sensitivities, which can affect inflammation and pain in fibromyalgia (26). Some patients concurrently took probiotics for microbiome optimization and glutamine to treat elevated intestinal permeability since emerging research has implicated the roles of both of these processes in fibromyalgia's pathogenesis (27, 28).

Several patients also partook in various forms of psychotherapy to treat emotional trauma or PTSD, which are commonly comorbid among patients with fibromyalgia (29, 30) and psychological therapies in persons with fibromyalgia are known to impact pain relief (31, 32). Furthermore, a biochemical mechanism involving neuroinflammation and glial cell inflammation has been observed in fibromyalgia (12), nociplastic pain (12) and PTSD (33) and appears to be modulated by LDN (10), suggesting that LDN may act on some of the underlying pathophysiological mechanisms connecting psychological trauma and fibromyalgia.

### Chronic pain diagnoses

The variable causes of widespread chronic pain may also affect the variability of treatment responses among publications on LDN. Fibromyalgia remains a clinical diagnosis, but it has been proposed that up to 11% of patients with fibromyalgia may be incorrectly diagnosed (34). For example, myofascial pain syndrome also involves widespread pain and tender points which resemble fibromyalgia, yet pain is generated by peripheral nociception (35) as opposed to the central sensitization implicated in fibromyalgia (1). While LDN has not specifically been studied in myofascial pain syndrome, other chronic pain syndromes involving central sensitization such as chronic regional pain syndrome (CRPS, 37) or opioid-induced hyperalgesia (36, 37) appear to respond to LDN whereas patients with pain generated from peripheral mechanisms such as inflammatory/rheumatological spondyloarthropathies (38) or osteoarthritis (39) do not. This observation deserves further investigation but suggests that the mechanism of LDN may be more specific to pain syndromes involving central sensitization and that not all patients with widespread chronic pain would be expected to respond to LDN.

## Limitations

Limitations in this case series include small sample size (*n* = 10), absence of a control group, extrapolation of baseline pain scores for patients who discontinued LDN early and limited generalizability, as patients tended to be motivated to seek treatments for pain relief, self-referred to the integrative medicine clinic and embraced a multi-modal pain management strategy.

## Conclusion

This case series illustrates the potential for LDN to provide substantial pain relief and other health benefits in patients with fibromyalgia with a favorable safety profile. We report important treatment responses to LDN as a first-line and second-line therapy at an integrative medicine clinic and highlight the importance of hormesis and custom dose titration for optimal therapeutic effects. Future research should continue to evaluate its utility as a treatment for pain syndromes involving central sensitization, such as fibromyalgia, but are advised to consider the utility of flexible patient specific dosing when designing protocols to account for a proposed mechanism involving hormesis. With growing evidence on the efficacy and safety of the use of LDN in fibromyalgia, this medication deserves to be considered as a treatment recommendation for pain management in fibromyalgia.

## Data Availability

The raw data supporting the conclusions of this article will be made available by the authors, without undue reservation.
